# Supplementary figures and images for: Circulating cell-free DNA from plasma undergoes less fragmentation during bisulfite treatment than genomic DNA due to low molecular weight
Source: PLoS One. 2019 Oct 25;14(10):e0224338. doi: 10.1371/journal.pone.0224338 (PMC6814277; doi:10.1371/journal.pone.0224338)

Figure 1

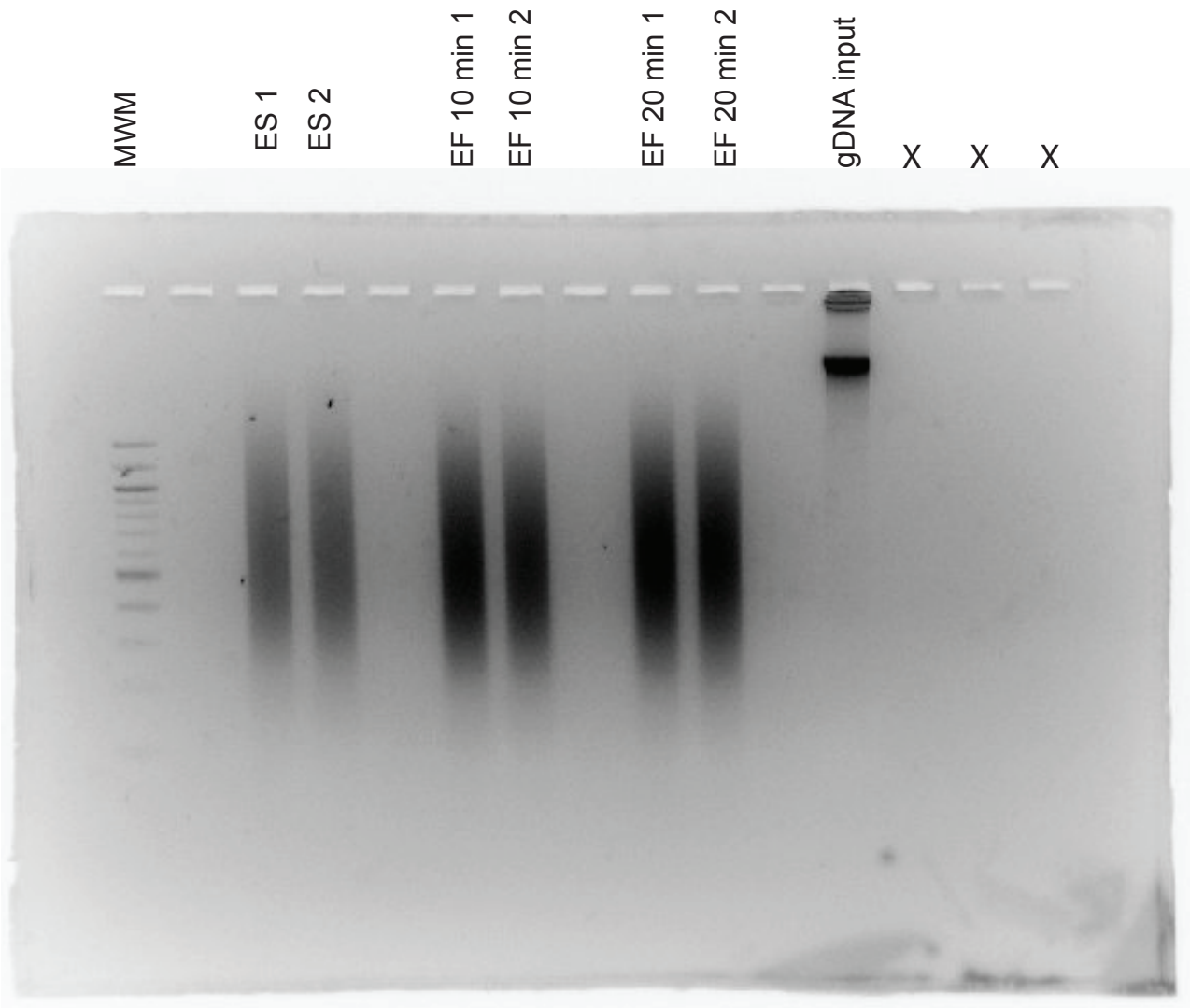

Figure 2

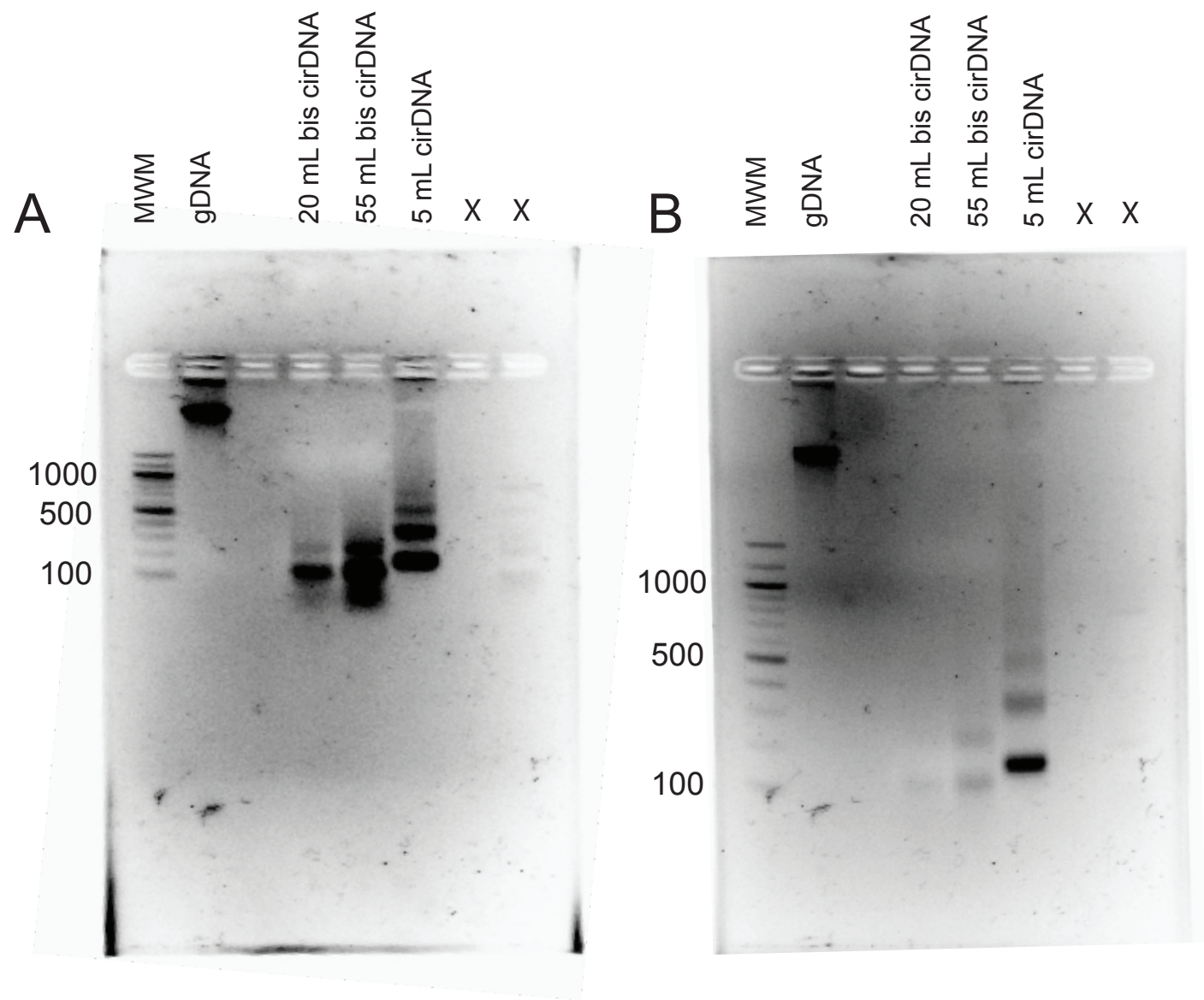

Figure 3

A

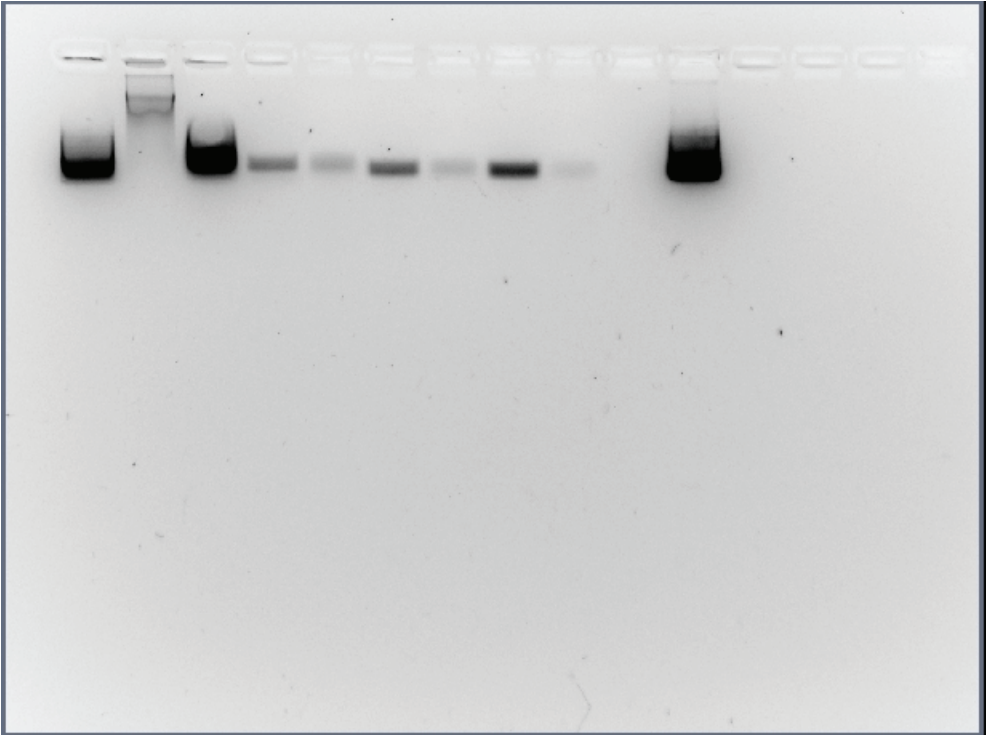

B

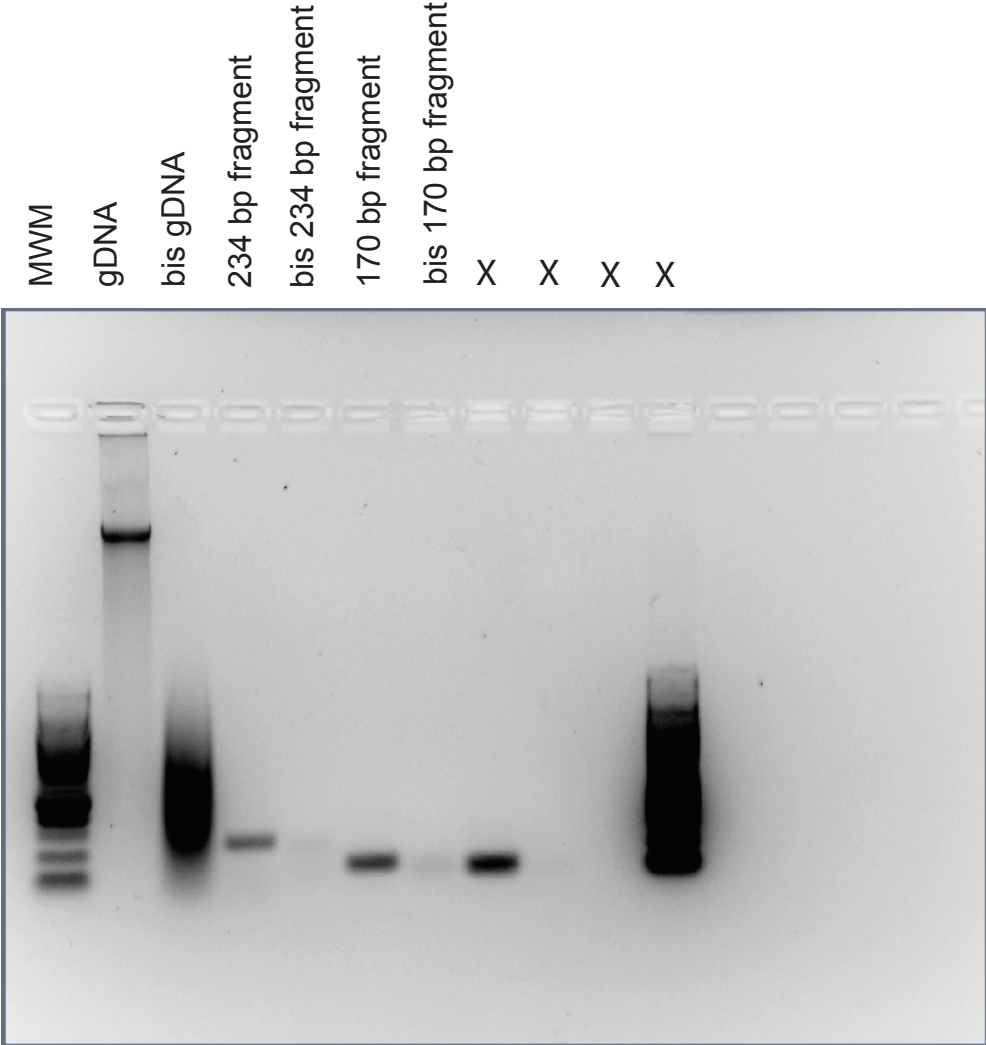

Supplement: S2 Fig — (PDF) [file pone.0224338.s002.pdf]
